# Prevalence and Associated Risk Factors of Intestinal Parasitic Infections among Pregnant Women Attending Antenatal Care in Yifag Health Center, Northwest Ethiopia

**DOI:** 10.1155/2021/7291199

**Published:** 2021-10-21

**Authors:** Destaw Damtie, Minichil Liyih

**Affiliations:** ^1^Department of Biology, Bahir Dar University, Bahir Dar, Amhara Regional State, Ethiopia; ^2^Abune Gorgorios School, Bahir Dar City Administration, Bahir Dar, Amhara Regional State, Ethiopia

## Abstract

**Background:**

A cross-sectional study was conducted from November 2019 to March 2020 to determine the prevalence and associated risk factors of intestinal parasitic infections (IPIs) among pregnant women attending antenatal care (ANC) at Yifag Health Center.

**Methods:**

The data were collected by a questionnaire interview technique and collecting the stool samples from each pregnant woman. Wet-mount and formol-ether concentration techniques were applied to identify the IPIs. Data were analyzed using SPSS, version 25, and *P*-values < 0.05 were considered statistically significant.

**Results:**

Out of the total 280 pregnant women who were selected using a simple random sampling technique, 277 participated in the questionnaire survey and gave stool samples (a response rate of 98.9%). The prevalence of IPIs among pregnant women was 53.4% (95% CI: 47.37, 59.42). *Taenia* species (18.1%) was the predominant parasite followed by *Giardia lamblia* (12.6%), *Entamoeba histolytica*/*dispar* (9.4%), hookworms (9%), *Ascaris lumbricoides* (4%), *Schistosoma mansoni* (3.2%), *Hymenolepis nana* (0.7%), *Strongyloides stercoralis* (0.4%), and *Enterobius vermicularis* (0.4%). Eating raw vegetables (AOR = 2.721; 95% CI: 1.266, 5.849; *P*=0.010) and poor personal hygiene (AOR = 4.015; 95% CI: 1.456, 11.07; *P*=0.007) were associated risk factors for *G. lamblia*, while eating raw meat (AOR = 2.477; 95% CI: 1.252, 4.902; *P*=0.009) for *Taenia* species infections. The prevalence of intestinal parasites was high and still a health burden to the pregnant women in the study area. We recommend avoiding eating raw meat, strengthening sanitation and hygiene programs, and routine deworming of pregnant mothers to reduce the burden of IPIs among pregnant women.

## 1. Introduction

Amebiasis, ascariasis, hookworm, and trichuriasis are among the top ten common intestinal parasitic infections (IPIs) [1]. IPIs caused by pathogenic helminth and protozoan species are endemic throughout the world, peculiarly in developing countries [2, 3]. About one-fourth of the world's population lives with intestinal parasites [4].

IPIs during pregnancy, if left untreated, can cause adverse effects for the mothers, fetuses, and newborns. Globally, the three most prevalent helminth infections among pregnant women are hookworm (19%), *A. lumbricoides* (17%), and *T. trichiura* (11%), and the three predominant protozoan infections are *Blastocystis* sp. (21%), *E. histolytica/dispar* (9%), and *Giardia* sp. (8%) [5].

In Ethiopia, the overall prevalence of IPIs among pregnant women is unknown. But separate studies have been conducted in various localities. For example, the overall prevalence of IPIs among pregnant women attending prenatal care at Felege Hiwot Referral Hospital, Bahir Dar, was 31.5% [6]; among pregnant women attending ANC at public health facilities in the Lalo Kile district, Oromia, Western Ethiopia, was 43.8% [7]; among pregnant women in West Gojjam Zone, Northwest Ethiopia, was 37.3% [8], and among pregnant women in the Wondo Genet district, Southern Ethiopia, was 38.7% [9].

The most predominant IPIs identified were *G. lamblia* (13.3%) followed by *E. histolytica*/*dispar* (7.8 %), hookworms (5.5 %), *A. lumbricoides* (2.9 %), *S. mansoni* (2.9 %), *S. stercoralis* (1.6 %), *Taenia* spp. (0.8 %), and *H. nana* (0.3 %) among pregnant women attending Felege Hiwot Referral Hospital [6]; hookworms (33.7%) followed by *A. lumbricoides* (7.3%) among pregnant women attending ANC at public health facilities in the Lalo Kile district, Oromia, Western Ethiopia [7]; hookworms (18.6%) followed by *E*. *histolytica*/*dispar* (15.2%) among pregnant women in West Gojjam Zone, Northwest Ethiopia [8]; and *A. lumbricoides* (24.9%) followed by hookworms (11.2%), *G. lamblia* (5.4%), *E. histolytica* (3.4%), *T. trichiura* (2.9%), and *S. mansoni* (2.3%) among pregnant women in the Wondo Genet district, Southern Ethiopia [9].

The distribution of IPIs depends on many factors such as low socioeconomic status, poor sanitation and personal hygiene, and lack of clean water [10]. The risk factors among pregnant women were being a farmer, walking barefooted, absence of proper handwashing after latrine [7], dwelling in a rural area, eating raw vegetables, improper use of toilets, poor environmental sanitation, the habit of soil eating, having irrigation practice, and lack of health education [8].

The aim of this study, therefore, was to determine the prevalence and associated risk factors of IPIs among pregnant women attending ANC at Yifag Health Center. The findings from this study would provide information about the status of IPIs and their associated risk factors among pregnant women in the Amhara region, serve as a springboard for Libokemkem District health officers and regional health officers, and will provide baseline information for further studies.

## 2. Methods

### 2.1. Study Area, Design, and Population

The location of Yifag Health Center is in Libokemkem District, South Gondar Zone, North West Ethiopia, at 12°5'0” latitude north and 37°44'0” longitude east. Its elevation ranges from 1829 to 1868 meters above sea level. It lies 77 km east of Bahir Dar, the capital city of the Amhara region, and 645 km northwest of Addis Ababa, the capital city of Ethiopia. It has a mean rainfall of 1330 ml and a mean temperature ranging from 22 to 30°C. There are five kebeles (lowest administrative units) whose residents are getting service from the health center. These kebeles harbor a total population of 198,374 (97,423 females, 100,951 males) [11]. Most of the residents of the study area are farmers who use mixed agriculture. The main cultivated crops are rice, maize, chickpea, oats (rye), and little irrigation practice of onion and garlic. Dug well and pipe waters are the sources of drinking water in the study area. But most people use river water for hygienic purposes. The sanitary facilities in the study area are open latrine systems. There are six health posts and one health center in the study area.

A health center-based cross-sectional study was conducted from 30 November 2019 to 07 March 2020. All the women visiting Yifag Health Center during the study period were the source population, while all the pregnant women were the study population.

### 2.2. Inclusion and Exclusion Criteria

The study included all pregnant women who were willing to give stool, sign informed consent, and did not take antiparasitic drugs for the last three weeks before screening.

### 2.3. Sample Size Determination and Sampling Technique

The sample size calculation used the single population proportion formula considering 95% CI and *P*=0.21 [12] and adding 10% assumption of nonresponse rate as follows:

(1) $$\begin{array}{c} n={\left(z_{\alpha /2}\right)}^{2}p\frac{\left(1-p\right)}{d^{2}}=255+10\%\left(255\right)=280, \end{array}$$

where *n* is the sample size, *z*_*α*/2_ = 1.96 for the standard scale of 95% level of confidence, and *d* is the marginal error, which is 0.05.

### 2.4. Collection of Stool Samples

The respondents were given clear instructions and provided with clean labeled collection cups along with applicator sticks. Each mother delivered about four grams of a fresh stool sample. The data collector registered the date of sampling, the name of each participant, and the age of each participant during data collection. The WHO guideline [13] was employed to process and examine a portion of each of the stool samples using direct wet-mount and formol-ether concentration techniques in the YHC laboratory class for parasitological examination.

### 2.5. Questionnaire Survey

The researchers developed a structured questionnaire first in the English language. The questionnaire incorporated issues about the sociodemography, water source, personal hygiene, latrine availability, residence, attitudes on IPIs, basic knowledge on common signs and symptoms of IPIs, pregnancy month, and interpregnancy of the respondents. Then, it was translated into the local language (Amharic) for interview. Before the interview, the questionnaires were pretested among thirty mothers. Then, we made the necessary adjustments based on the feedback. The data collectors interviewed the respondents during stool collection. We translated the responses back into English for data analysis.

### 2.6. Direct Microscopy (Wet Mount) Procedure

In the wet mount, a fresh stool sample of each participant (about 2 mg) was placed on a glass slide with a wooden applicator, emulsified with a drop of physiological saline (0.85%) for diarrheic and semisolid samples. For formed stools, iodine was used. Then, covered with cover slide and examined for the presence of motile intestinal parasites and trophozoites under a microscope using first X10 objectives and then X40 objectives [14].

### 2.7. Formol-Ether Concentration Technique

A portion of each stool sample was used for the detection of parasitic ova and protozoan cysts using the formol-ether concentration technique. About 2 grams of each stool sample was first emulsified with three to four ml of 10% formol saline. This was mixed thoroughly and passed through the gauze [14]. Three to four ml of diethyl ether was added and mixed by inverting and intermittent shaking for one minute and then centrifuged at 3,000 rpm for five minutes. After centrifugation, the supernatant (layers of ether, debris, and formol saline) was discarded by a pipette and the sediment (containing the parasites at the bottom of the test tube) was resuspended in formol saline. The sediment was examined microscopically under 10X by 10X and 10X by 40X magnifications for the presence of any parasitic organisms [14]. To maintain the reliability of the study findings, the specimen was reexamined at the end by an experienced laboratory technologist who was blind for the first examination result.

### 2.8. Variables

The prevalence of the parasitic infections was the dependant variable, while associated risk factors, sociodemographic factors (age, residence, educational level, family size, and religion), socioeconomic factors (occupation, access to clean water, access to the toilet, knowledge about IPIs), environmental factors (source of water), and behavioral factors (hand wash before food and after toilet, eating raw food, personal hygiene, shoe-wearing habit, waste discharge habit, fingernail status) were independent variables.

### 2.9. Data Analysis

Statistical Package for Social Sciences (SPSS) version 25 was used to analyze the collected data. Descriptive statistics such as frequency, percentage, mean, and range were determined for each intestinal parasite. Binary logistic regression was used to measure the strengths of association between the prevalence of infection and the risk factors using the odds ratio. In the modeling process, a univariate analysis was first carried out with less than a 0.25 level of significance to select the candidate variables for multivariate analysis. The variables, significant at a *P*-value of <0.25 in the univariate analysis, were then included in the multivariate analysis [15]. Values were considered significant at *P* < 0.05.

### 2.10. Ethical Consideration

Ethical clearance was obtained from the Ethical Review Committee of the Science College, Bahir Dar University, before data collection (S1_File). A letter describing the objective of the study was written to the Libokemkem Health Office and Yifag Health Center. The researcher obtained consent from the study participants after explaining the purposes and the procedures of the study. The laboratory test and the questionnaires were conducted with strict privacy and confidentiality. The pregnant women whose test results are positive were given standard drugs free of charge.

## 3. Results

### 3.1. Sociodemographic Characteristics of the Study Participants

A total of 277 (99%) pregnant women gave stool for intestinal parasitic examination and filled the questionnaires. The mean age of the study subjects was 26.6 years. Two hundred four (73.6%) were farmers, 22 (7.9%) housewives, 9 (3.3%) government employees, and 42 (15.2%) merchants. Two hundred thirteen (76.9%) and 64 (23.1%) of the participants had family sizes of ≤5 and >5, respectively. The educational status of the participants was illiterate (63.2%), primary school (20.9%), secondary school (10.5%), and diploma and above (5.4%) (Table 1).

### 3.2. Hygienic Characteristics of the Respondents

The proportions of the respondents for hygienic characteristics were access to protected water (93.1%), access to toilet (6.9%), personal hygiene (good/poor: 33.2%/66.8%), awareness about parasitic infections (good/poor: 19.5%/80.5%), handwashing habit (before feeding = 99.6%, after defecation = 58.8%, not at all = 41.2%), habit of wearing shoes (regularly = 54.2%, not at all = 45.8%), waste disposal mechanism (burry/burn = 9.0%, open field disposal = 91.0%), fingernail status (trimmed = 58.5%, untrimmed = 41.5%), and raw meat/vegetable feeding habit (40.1%/45.8%) (Table 2).

### 3.3. Prevalence of IPIs in the Study Population

Two species of protozoa and seven species of intestinal helminths were identified from the stool samples of the study participants. The overall prevalence of IPIs was 53.4%. *Taenia* species (18.1%) and *G. lamblia* (12.6%) were the two top and *E. vermicularis* (0.4%) and *S. stercoralis* (0.4%) were the least prevalent IPIs. The prevalence of protozoa and helminths was 22.0% and 35.7%, respectively. The rates of the single, double, and triple infections were 49.5%, 3.6%, and 0.4%, respectively (Table 3). Most of the double infections occurred between the *Tania* species and others, and triple infections occurred among *E. histolytica/dispar*, *G. lamblia,* and hookworms.

### 3.4. Factors Associated with IPIs among Pregnant Women Visiting YHC for ANC

In both the univariate and multivariate logistic regressions, no specific risk factors were observed for *E. histolytica/dispar*, hookworms, *A. lumbricoides,* and *S. mansoni* (*P* > 0.05). Risk factors were identified to *G. lamblia* and *Taenia* species (*P* < 0.05) (Table 4). The multivariate logistic regression showed that mothers who had the habit of eating raw vegetables were 2.721 times more infected by *G. lamblia* (AOR = 2.721; 95% CI: 1.266, 5.849; *P*=0.010) than their counterparts, and mothers who had poor personal hygiene were four times more likely infected (AOR = 4.015; 95% CI: 1.456, 11.07; *P*=0.007) by *G. lamblia* than those with good personal hygiene (Table 4). Furthermore, mothers who had the habit of eating raw meat were 2.5 times (AOR = 2.477; 95% CI: 1.252, 4.902; *P*=0.009) more likely to be infected by the *Taenia* species than their contemporaries.

## 4. Discussion

The overall prevalence of IPIs in this study was 53.4% (95% CI: 47.4, 59.4). It was comparable with previous studies conducted in North Ethiopia (51.5%) [16] and Nigeria (56.8%) [17]. But it was higher than the findings from the Gilgel Gibe Dam area, Southwest Ethiopia (11.6%) [18]; Lalo Kile district, Western Ethiopia (43.8%) [7]; Dembiya, Northwest Ethiopia (25.8%) [19]; Colombia (41%) [20]; Nepal (35%) [2]; and Kenya (13.8%) [21] and lower than the studies conducted in Mecha District, Northwest Ethiopia (70.6%) [22]; Papua New Guinea (81.0%) [23]; and Venezuela (73.9%) [23, 24]. These variations might be due to the differences in the sociodemographic factors, lack of awareness on the prevention of parasitic infections, personal hygiene, eating uncooked food, not trimming fingernails, poor waste disposal, bare footedness, lack of clean water, and environmental factors.

The most prevalent IPI in this study was the *Taenia* species (18.1%). A lower prevalence of *Taenia* species was reported from Arba Minch Town, Ethiopia (0.6%) [25]; Bahir Dar, Northwest Ethiopia (0.8%) [6]; East Wollega, Ethiopia (1.3%) [26]; and Iran (0.014%) [27]. This difference may be due to the differences in the habit of eating uncooked meat, altitude, open defecation, and poor awareness in the prevention of this parasite [28].

*G. lamblia* was the second most prevalent parasite (12.6%) among the study participants. This finding was in line with previous studies conducted in Southern Ethiopia (12.6%) [29]; Bahir Dar, Northwest Ethiopia (13.3%) [6]; and Venezuela (14.1%) [24]. It was lower than the prevalence in studies conducted in Northwest Ethiopia (19.2%) [30] and Papua New Guinea (39%) [23]. But it was higher than studies conducted in Wolayita Sodo Town, Southern Ethiopia (3.6%) [31]; Lalo Kile district, Western Ethiopia (0.9%) [7]; and Wondo Genet district, Southern Ethiopia (5.4%) [9]. Accessibility of safe water, open field defecation, and variations in hand wash implementation might bring such differences [32].

*E. histolytica/dispar* infection among the study participants was 9.4%. It was consistent with studies conducted in the Gamo area, Southern Ethiopia (11.4%) [33]; Bahir Dar, Northwest Ethiopia (7.8%) [6]; and Venezuela (12.0) [24]. But it was lower than the reports from Nigeria (18.9% [17], 2010) and Papua New Guinea (43%) [23] and higher than the findings from Southwest Ethiopia (5.5%) [34]; Wondo Genet district, Southern Ethiopia (3.4%) [9]; and Tanzania (0.7%) [35]. Accessibility of safe water, open field defecation, variations in handwashing habits before food and after toilet, eating raw vegetables, contact with night soil, sanitation problems, and environmental and climate factors might bring such differences [36].

The level of hookworm in the present study was 9.0%. This finding was in agreement with the reports from Southern Ethiopia (9.9%) [29]; Wondo Genet district, Southern Ethiopia (11.2%) [9]; and Venezuela (8.1%) [24]. However, this prevalence was lower than that of the study conducted in Northwest Ethiopia (20%) [12]; the Gilgel Gibe Dam area, Southwest Ethiopia (29.4%) [18]; Lalo Kile district, Western Ethiopia (33.7%) [7]; the Maytsebri primary hospital, North Ethiopia (39.96%) [16]; Uganda (40.5%) [37]; and Nigeria (44.4%) [38]. But it was higher than the reports from Kenya (3.92%) [21] and Bahir Dar, Northwest Ethiopia (5.5%) [6]. This difference might be due to the differences in geography, shoe-wearing habits, level of income, and agricultural practices [7].

The prevalence of *A. Lumbricoides* in the present study was 4%. It was comparable with the results of the reports from Wolayita Sodo Town, Southern Ethiopia (5.5%) [31]; Bahir Dar, Northwest Ethiopia (2.9%) [6]; and Kenya (6.5%) [21]. But it was higher than the findings in Arba Minch Town, Ethiopia (0.3%) [25] and in Ghana (0.9%) [39] and lower than results from Lalo Kile district, Western Ethiopia (7.3%) [7]; the Gilgel Gibe Dam area, Southwest Ethiopia (15%) [18]; the Maytsebri primary hospital, North Ethiopia (12.7%) [16]; Wondo Genet district, Southern Ethiopia (24.9%) [9]; Mecha District, Northwest Ethiopia (32.2%) [22]; Nigeria (52.2%) [38]; and Venezuela (57.0) [24]. The observed difference might be due to environmental conditions and environmental sanitation problems, differences in eating raw vegetables, lack of handwashing, agricultural practices, waste disposal habit, lack of clean waters, and open defecations [22, 40].

The prevalence of *S. mansoni* was 3.2%. It was comparable with studies conducted in Bahir Dar, Northwest Ethiopia (2.9%) [6], and Jimma, Southwest Ethiopia (3.6%) [34]. However, it was higher than the finding from Northwest Ethiopia (2.2%) [12]; Wondo Genet district, Southern Ethiopia (2.3%) [9]; and Wolayita Sodo Town, Southern Ethiopia (0.6%) [31]. But it was lower than that of the studies conducted in Mecha District, Northwest Ethiopia (17.4%) [22], Waja-Timuga, District of Alamata, Northern Ethiopia (73.9%) [41]; Cameroon (28.01%) [42]; and Uganda (36.4%) [37]. The variations in *S. mansoni* infection could be due to the differences in the geographical areas, environmental pollution with urine and feces, the habit of crossing and bathing in river waters, eating uncooked vegetables and foods, and cercariae-infested water sources [41].

The prevalence of *H. nana* in this study was 0.7%. This prevalence agreed with the findings from Gondar Town, Northwest Ethiopia (0.5%) [43], and Bahir Dar, Northwest Ethiopia (0.3%) [6]. However, the findings of studies reported from the Gilgel Gibe Dam area, Southwest Ethiopia (1.6%) [18]; East Wollega, Oromia, Ethiopia (1.6%) [26]; and Baghdad/Iraq (6.67%) [44] were higher than the current finding. These variations might be due to the variations in the maintenance of good personal hygiene and sanitary improvements, consumption of contaminated food and water, and rodent control [45].

The prevalence of *S. stercoralis* in the current finding was 0.4%, which was in line with the study conducted in East Wollega, Ethiopia (0.3%) [26], and Lalo Kile district, Western Ethiopia (0.3%) [7]. But it showed a lower prevalence of *S. stercoralis* than that found in studies conducted in Bahir Dar, Northwest Ethiopia (1.6%) [6]; Mecha District, Northwest Ethiopia (6.4%) [22]; Nigeria (1.3%) [38]; Papua New Guinea (3%) [23]; and Venezuela (3.3%) [24]. These variations may be accounted for by the differences in contamination of the soil with feces, walking barefoot, open latrine system, and environmental sanitation [46].

In this study, the prevalence of *E. vermicularis* was 0.4%. It was consistent with the study conducted in the Gilgel Gibe Dam area, Southwest Ethiopia (0.3%) [18]. However, it was lower than that found in other studies reported from Nigeria (3.5%) [47], Kenya (4.8%) [21], and Venezuela (6.3%) [24] and far lower than Iraq (32.9%) [48]. These variations in the prevalence of *E. vermicularis* among studies could be due to the differences in environmental sanitation, parasitological methods used during the study, and maintenance of personal and community hygiene such as frequent handwashing, fingernail cleaning, regular bathing, and washing of nightclothes and bed lining [45].

High family size, unsafe and inadequate provision of water, unhygienic living conditions, absence or improper utilization of latrine, not washing hands after toilet, and the habit of walking barefoot are significantly associated with IPIs [7,14,22,49]. But these factors did not found to be statistically associated with IPIs in the present study.

In the present study, we found IPIs statistically associated with eating raw meat with the odds of 1.66 (AOR = 1.66; 95% CI: 1.03, 2.67, *P*=0.036). This finding is congruent with the study finding around Lake Ziwai, Ethiopia [50].

One of the risk factors for *G. lamblia* was eating raw vegetables with the odds of 2.72 (AOR = 2.72; 95% CI: 1.27, 5.85; *P*=0.010*P* = 0.010). It is consistent with the study finding in East Wollega, Ethiopia [26]. The other associated risk factor was poor personal hygiene. Mothers with poor personal hygiene were 4.02 times (AOR = 4.015; 95% CI: 1.46, 11.07; *P*=0.007) more infected than their counterparts. This report is in agreement with the findings from Jawi Town, Northwest Ethiopia [51], and Goiânia, Goiás State, Brazil [36].

*Taenia* species was significantly associated with eating raw meat. Mothers who ate raw meat were 2.26 times more infected than their counterparts (AOR = 2.26; 95% CI: 1.13, 4.55, *P*=0.02). This finding was supported by the finding from around Lake Ziwai, Ethiopia [50]. However, other identified IPIs were not significantly associated with any of the potential risk factors.

## 5. Conclusions

This study indicated that there was a high prevalence of IPIs among pregnant women in the selected area. The most common detected intestinal parasites were *Taenia* species followed by *G. lamblia*, *E. histolytica/dispar,* and hookworms. Eating raw meat was an associated risk factor for IPIs. Eating raw vegetables and poor personal hygiene were predictors of *G. lamblia,* and eating raw meat was an associated risk factor for *Taenia* species. So, avoiding eating raw meat and vegetables, making and strengthening sanitation and hygiene programs, creating awareness about IPIs, making closed toilets, and routine deworming of mothers before pregnancy and on second and third trimesters are recommended.

### 5.1. Limitation

Use of single-season data, use of only wet mount and formol-ether concentration techniques to identify IPIs, and taking stool samples only in the daytime which may affect *E. vermicularis* load were the limitations of this study.

## Figures and Tables

**Table 1:** Sociodemographic profile of pregnant women at Yifag Health Center, Northwest Ethiopia (*N* = 277), from November 2019 to March 2020.

Variable	Categories	Frequency	Percent
Age group	18–30	224	80.9
	31–45	53	19.1

Residence	Around Yifag	43	15.5
	Bura	92	33.2
	Ginaza	19	6.9
	Shina	28	10.1
	Yifag	95	34.3

Religion	Muslim	17	6.1
	Orthodox	260	93.9

Occupation	Agriculture	204	73.6
	Government employed	9	3.2
	Housewives	22	7.9
	Merchant	42	15.2

Educational level	Diploma and above	15	5.4
	Illiterate	175	63.2
	Primary	58	20.9
	Secondary	29	10.5

Family no.	≤5	213	76.9
	>5	64	23.1

**Table 2:** Practice of pregnant women related to personal and environmental hygiene taking ANC at Yifag Health Center, Northwest Ethiopia (*N* = 277), November 2019 to March 2020.

Variable	Categories	Frequency	Percent
Waste disposal	Burring or burning	25	9.0
	Open field	252	91.0

Have toilet	No	206	74.4
	Yes	71	25.6

Type of toilet	Private	31	11.2
	Open field	209	75.5
	Public	37	13.4

Source of water	Pipe	258	93.1
	Unprotected	19	6.9

Handwashing before feeding	No	1	0.4
	Yes	276	99.6

Handwashing after toilet	No	163	58.8
	Yes	114	41.2

Fingernail status	Trimmed	162	58.5
	Untrimmed	115	41.5

Eating raw meat	No	166	59.9
	Yes	111	40.1

Eating raw vegetables	No	150	54.2
	Yes	127	45.8

Personal hygiene	Good	92	33.2
	Poor	185	66.8

Shoe wearing	No	127	45.8
	Yes	150	54.2

Transmission and prevention knowledge of IPI	No	223	80.5
	Yes	54	19.5

**Table 3:** Prevalence of IPIs among pregnant women (*N* = 277) attending ANC in Yifag Health Center, Northwest Ethiopia, November 2019 to March 2020.

Parasite	Number women infected	Percent
Protozoa		
*E. histolytica/dispar*	26	9.4
*G. lamblia*	35	12.6
Total	61	22

Helminths		
*A. lumbricoides*	11	4.0
*Hookworm*	25	9.0
*H. nana*	2	0.7
*S. mansoni*	9	3.2
*Taenia* spp.	50	18.1
*E. vermicularis*	1	0.4
*S. stercoralis*	1	0.4
Total	99	35.7

Single and multiple infections		
Single infections	137	49.5
Double infections	10	3.6
Triple infections	1	0.4

The overall prevalence of IPIs	148	53.4

**Table 4:** Univariate and multivariate logistic regression analysis of potential risk factors associated with IPIs among pregnant women in Yifag Health Center, Northwest Ethiopia, November 2019 to March 2020.

Risk factors	Categories	Total	Negative no. (%)	Positive no. (%)	Univariate logistic regression		Multivariate logistic regression	
					COR (95% CI)	*P*-value	AOR (95% CI)	*P*-value
*E. histolytica/dispar*								
Type of toilet	Private	31	25 (80.6)	6 (19.4)	1	0.149	1	0.149
	Open field	209	192 (91.9)	17 (8.1)	0.369 (0.133, 1.023)		0.369 (0.133, 1.023)	
	Public	37	34 (91.9)	3 (8.1)	0.368 (0.084, 1.613)		0.368 (0.084, 1.613)	
*G. lamblia*								
Fingernail status	Trimmed	162	146 (90.1)	16 (9.9)	1	0.104	—	0.111
	Untrimmed	115	96 (83.5)	19 (16.5)	1.806 (0.885, 3.685)		1.831 (0.870, 3.853)	
Eating raw vegetables	No	150	136 (90.7)	14 (9.3)	1	0.076	1	0.010^*∗*^
	Yes	127	106 (83.5)	21 (16.5)	1.925 (0.935, 3.963)		2.721 (1.266, 5.849)	
Personal hygiene	Good	92	87 (94.6)	5 (5.4)	1	0.015^*∗*^	1	0.007^*∗*^
	Poor	185	155 (83.8)	30 (16.2)	3.368 (1.261, 8.996)		4.015 (1.456, 11.07)	
*A. lumbricoides*								
Hand wash after toilet	No	163	159 (97.5)	4 (2.5)	0.385 (0.110, 1.346)	0.135	0.827 (0.186, 3.676)	0.803
	Yes	114	107 (93.9)	7 (6.1)	1		1	
Waste disposal	Burial/burning	25	22 (88.0)	3 (12.0)	1	0.046^*∗*^	1	0.372
	Open field	252	244 (96.8)	8 (3.2)	0.240 (0.059, 0.972)		0.487 (0.100, 2.362)	
Fingernail status	Trimmed	162	158 (97.5)	4 (2.5)	1	0.141	1	0.140
	Untrimmed	115	108 (93.9)	7 (6.1)	2.560 (0.732, 8.959)		2.618 (0.729, 9.409)	
Knowledge about IPIs	No	223	218 (97.8)	5 (2.2)	0.183 (0.054, 0.626)	0.007^*∗*^	0.240 (0.057, 1.005)	0.051
	Yes	54	48 (88.9)	6 (11.1)	1		1	
Hookworm								
Age group	18–30	224	206 (92)	18 (8)	1	0.242	1	0.383
	31–45	53	46 (86.8)	7 (13.2)	1.742 (0.687, 4.413)		1.520 (0.593, 3.896)	
Eating raw meat	No	166	154 (92.8)	12 (7.2)	1	0.206	1	0.204
	Yes	111	98 (88.3)	13 (11.7)	1.702 (0.746, 3.883)		1.713 (0.746, 3.932)	
Knowledge about IPIs	No	223	200 (89.7)	23 (10.3)	2.990 (0.683, 13.092)	0.146	2.871 (0.648, 12.727)	0.165
	Yes	54	52 (96.3)	2 (3.7)	1		1	
*S. mansoni*								
Occupation	Agriculture	204	200 (98.0)	4 (2.0)	0.400 (0.071, 2.259)	0.083	—	0.518
	Housewives	22	19 (86.4)	3 (13.6)	3.158 (0.486, 20.503)		—	
	Merchant	42	40 (95.2)	2 (4.8)	—		—	
	Government	9	9 (100.0)	0 (0.0)	1		1	
Hand wash after toilet	No	163	160 (98.2)	3 (1.8)	0.338 (0.083, 1.379)	0.130	0.919 (0.132, 6.389)	0.932
	Yes	114	108 (94.7)	6 (5.3)	1		1	
Shoe wearing	No	127	125 (98.4)	2 (1.6)	0.327 (0.067, 1.602)	0.168	0.709 (0.104, 4.841)	0.725
	Yes	150	143 (95.3)	7 (4.7)	1		1	
Toilet	Absent	206	201 (97.6)	5 (2.4)	0.417 (0.109, 1.597)	0.202	—	0.999
	Present	71	67 (94.4)	4 (5.6)	1		1	
Type of toilet	Private	31	30 (96.8)	1 (3.2)	1	0.233	—	0.745
	Open	209	204 (97.6)	5 (2.4)	0.735 (0.083, 6.511)		—	
	Public	37	34 (91.9)	3 (8.1)	2.647 (0.261, 26.823)		2.614 (0.225, 30.400)	
Fingernail status	Trimmed	162	155 (95.7)	7 (4.3)	1	0.248	1	0.468
	Untrimmed	115	113 (98.3)	2 (1.7)	0.392 (0.080, 1.922)		0.537 (0.100, 2.879)	
Personal hygiene	Good	92	86 (93.5)	6 (6.5)	1	0.045^*∗*^	1	0.231
	Poor	185	182 (98.4)	3 (1.6)	0.24 (0.058, 0.967)		0.341 (0.059, 1.98)	
*Taenia* species								
Residence	Around Yifag	43	32 (74.4)	11 (25.6)	3.285 (1.245, 8.664)	0.033^*∗*^	2.051 (0.485, 8.668)	0.510
	Bura	92	70 (76.1)	22 (23.9)	3.003 (1.300, 6.937)		1.616 (0.410, 6.366)	
	Ginaza	19	18 (94.7)	1 (5.3)	0.531 (0.063, 4.456)		0.332 (0.030, 3.710)	
	Shina	28	21 (75.0)	7 (25.0)	3.185 (1.064, 9.539)		1.691 (0.344, 8.309)	
	Yifag	95	86 (90.5)	9 (9.5)	1		1	
Religion	Muslim	17	16 (94.1)	1 (5.9)	1	0.208	1	0.718
	Orthodox	260	211(81.2)	49 (18.8)	3.716 (0.481, 28.692)		0.616 (0.044, 8.542)	
Occupation	Agriculture	204	161 (78.9)	43 (21.1)	0.534 (0.128, 2.224	0.057	0.108 (0.013, 0.904)	0.063
	Housewives	9	6 (66.7)	3 (33.3)	0.200 (0.027, 1.490)		0.117 (0.013, 1.045)	
	Merchant	22	20 (90.9)	2 (9.1)	0.100 (0.014, 0.727)		0.054 (0.006, 0.489)	
	Government	42	40 (95.2)	2 (4.8)	1		1	
Hand wash after toilet	No	163	127 (77.9)	36 (22.1)	2.025 (1.035, 3.959)	0.039^*∗*^	1.442 (0.613, 3.395)	0.402
	Yes	114	100 (87.7)	14 (12.3)	1		1	
Shoes wearing	No	127	96 (75.6)	31 (24.4)	2.226 (1.187, 4.176)	0.013^*∗*^	1.595 (0.741, 3.435)	0.233
	Yes	150	131 (87.3)	19 (12.7)	1		1	
Toilet	Absent	206	163 (79.1)	43 (20.9)	2.412 (1.031, 5.640)	0.042^*∗*^	0.513 (0.040, 6.549)	0.608
	Present	71	64 (90.1)	7 (9.9)	1		1	
Type of toilet	Private	31	29 (93.5)	2 (6.5)	1	0.085	1	0.613
	Open field	209	165 (78.9)	44 (21.1)	3.867 (0.888, 16.834)		4.795 (0.212, 108.62)	
	Public	37	33 (89.2)	4 (10.8)	1.758 (0.300, 10.310)		1.675 (0.223, 12.550)	
Eating raw meat	No	166	144 (86.7)	22 (13.3)	1	0.012^*∗*^	1	0.009^*∗*^
	Yes	111	83 (74.8)	28 (25.2)	2.208 (1.187, 4.106)		2.477 (1.252, 4.902)	
Eating raw vegetables	No	150	119 (79.3)	31 (20.7)	1	0.220	1	0.355
	Yes	127	108 (85)	19 (15)	0.675 (0.361, 1.265)		0.708 (0.341, 1.471)	
Personal hygiene	Good	92	81 (88)	11 (12)	1	0.066	1	0.595
	Poor	185	146 (78.9)	39 (21.1)	1.967 (0.955, 4.050)		1.295 (0.499, 3.358)	

^*∗*^ = statically significant at *P* < 0.05.

## Data Availability

All the data generated or analyzed during this study are included in this published article (and its supplementary information files).

## Supplementary Materials

Supplementary Materials S1_File: ethical clearance letter.
