# Supplementary material for: Prevalence and Associated Risk Factors of Intestinal Parasitic Infections among Pregnant Women Attending Antenatal Care in Yifag Health Center, Northwest Ethiopia
Source: Can J Infect Dis Med Microbiol. 2021 Oct 21;2021:7291199. doi: 10.1155/2021/7291199 (PMC8553486; doi:10.1155/2021/7291199)
Supplement: Supplementary Materials — S1_File: ethical clearance letter. [file 7291199.f1.pdf]

ሳይንስ ኮሌጅ
የድህረ ምረቃ፣ምርምርና ማህበረሰብ
አገልግሎት ም/ዲን
ባሕር ዳር ዩኒቨርሲቲ
ባሕር ዳር - ኢትዮጵያ

Wisdom at the source of the Blue Nile

Science College
The Graduate, Research
& Community Services V/Dean
Bahir Dar University
Bahir Dar – Ethiopia

✉ **79**

251 (582) 226 6597
ፋክስ Fax: 251 (582) 220- 20- 25

e-mail: negatassie@yahoo.com
website: www.bdu.edu.et

ቁጥር: **PGRCSVD/103/2012**

ቀን: 08/07/2012

Ethical Clearance Approval Form

Applicant's Name: Minichil Liyih

Research Title	Prevalence of intestinal parasites associated risk factors and its association with anaemia among pregnant women attending antenatal care in Yifag Heath Center
Researcher (s) Name (s)	Minichil Liyih

Thank you for submitting your application for ethical clearance, which was considered at the College of Science Research Ethics Committee meeting on 17 March 2020. The committee has reviewed your ethical application, issues pertaining to participants, consent form, debriefing, and relevant questionnaires.

The researcher should keep the confidentiality of the identity of research participants and data that will be obtained from them. Any serious adverse events or significant changes which occur in connection with this study and /or which may alter its ethical consideration must be reported immediately to the committee for a possible ethical amendment.

We are therefore pleased to inform you that the College's Ethical Clearance Committee has approved your study from an ethical point of view.

With kind regards

Dr. Tsegaye Kassa Gogie
P.G.R.CS. Vice Dean

Office of Research & Community Services
Bahir Dar University College of Science

CC//

- Dean office
- The Graduate, Research and Community Services V/Dean
- Biology Department

College of Science
